# Prevalence of suicidal behavior in bipolar type 1 patients

**DOI:** 10.1192/j.eurpsy.2024.1630

**Published:** 2024-08-27

**Authors:** F. Tabib, S. Omri, R. Bouaziz, I. Gassara, R. Feki, L. Zouari, J. Ben Thabet, N. Charfi, M. Maalej, N. Smaoui, M. M. Bouali

**Affiliations:** ^1^department of Psychiatry C, UHC Hedi Chaker, Sfax, Tunisia

## Abstract

**Introduction:**

The prevalence of suicidal behavior in individuals diagnosed with Bipolar Disorder Type 1 is a topic of great concern within the field of psychiatry and mental health research. Bipolar Disorder Type 1 is characterized by extreme mood fluctuations that can contribute to a heightened risk of suicidal ideation, attempts, and completions in affected individuals.

**Objectives:**

- To examine the socio-demographic and clinical profiles of Bipolar Type 1 patients admitted to the “C” psychiatry department at Hedi Chaker Hospital in Sfax, Tunisia.
- To identify and understand the factors associated with suicidal behavior in this population.

**Methods:**

We conducted a retrospective descriptive and analytic study of hospitalized patients suffering from bipolar disorder type 1 in the psychiatry department “C”, Hedi Chaker Hospital, Sfax Tunisia from 2021 to 2023. Socioeconomic data and clinical profiles of patients were collected from archived files.

**Results:**

The total number of patients was 98, with an average age of 36.74 ± 12.3 years. The majority were single (67%), living with their families (76.5%), jobless (45.9%), and receiving family support (94.9%). In terms of psychoactive substance use, 81.6% have used tobacco, 46.9% have used alcohol, and 34.7% have used cannabis.

Concerning family history, 55% of patients had at least one family member being treated for a mood disorder. Among them, 7.1% had attempted suicide, and 6.1% had died by suicide.

Concerning the clinical profile of the study population, 28.6% had a personal somatic history. The diagnosis of bipolar disorder was made at the age of 27.52±8.6 years. 11.2% had a comorbid personality disorder with bipolar disorder.

The majority of patients were on antipsychotics (95.9%), 84.7% were using mood stabilizers, 33.7% were prescribed anxiolytics, and only 4.1% were on antidepressants. Treatment compliance was poor in 61.2% of cases and 63.3% of patients had a poor insight.

Ten percent of these patients had attempted suicide, 50% during a depressive episode, 50% occurring during a depressive episode, 30% during a manic episode, and 40% of attempts were related to discontinuation of treatment. 3.1% had used hanging, and 3.1% had engaged in voluntary drug ingestion as a method of self-harm. None of the suicide attempts necessitated intensive care hospitalization, but 60% of the individuals were admitted to psychiatric care. There was a statistically significant correlation between suicide attempts and a family history of suicide (p=0.049).

**Conclusions:**

Bipolar patients face a heightened risk of suicide, which is closely tied to the distinctive attributes of the disorder, including biological factors, thymic decompensation, and psychological aspects. Consequently, managing their condition necessitates a tailored approach, demanding ongoing vigilance for individuals diagnosed with bipolar disorder.

**Disclosure of Interest:**

None Declared

